# Clinical Variables Affecting The Pregnancy Rate of Intracervical Insemination Using Cryopreserved Donor Spermatozoa: A Retrospective Study in China

**Published:** 2012-12-17

**Authors:** Xiao-Jun Chen, Li-Ping Wu, Hai-Lian Lan, Li Zhang, Yi-Min Zhu

**Affiliations:** Department of Reproductive Endocrinology, Women’s Hospital, School of Medicine, Zhejiang University, Hangzhou, Zhejiang, China

**Keywords:** Semen Donor, Artificial Insemination, Pregnancy Rate

## Abstract

**Background:**

The aim of this study was to investigate whether several clinical variables can affect the pregnancy rate of intracervical insemination (ICI) using cryopreserved donor spermatozoa.

**Materials and Methods:**

In this retrospective study, age, years of infertility, cervicitis, urinary luteinizing hormone (LH) surge, insemination number, uterus position, endometrial thickness and morphology, maximal follicle diameter, and the number of dominant follicles on the day of human chorionic gonadotropin (HCG) administration were retrospectively analyzed in 501 women who underwent their first ICI cycle using cryopreserved donor spermatozoa.

**Results:**

Increased age, length of infertility (>5 years), retroverted uterine position, and endometrial thickness (<7 mm or >14 mm) were associated with lower rates of pregnancy.

**Conclusion:**

In older women with infertile periods longer than five years, especially those with a retroverted uterus, intrauterine insemination (IUI) combined with ovarian stimulation should be recommended. *in vitro* fertilization with donor spermatozoa (IVFD) should be offered earlier to achieve a much higher success rate.

## Introduction

In the approximately 15% of couples that are affected by infertility, almost half of those cases are attributed to male cause (1 ). Recent developments in assisted reproductive techniques such as intracytoplasmic sperm injection (ICSI) and pre-implantation genetic diagnosis (PGD) have allowed most infertile men to regain fertility; however, parents with genetic defects give birth to progeny with a higher incidence of genetic abnormalities. Therefore, couples in which the male has severe oligo-, terato-, asthenozo-, or azoospermia; has undergone unsuccessful medical and surgical treatment to improve semen quality; or a failed ICSI treatment, will turn to choose artificial insemination by donor (AID) to have a healthy child.

In recent years, many studies have investigated AID techniques in order to improve the pregnancy rate. We know that intrauterine insemination (IUI) results in a higher pregnancy rate than intracervical insemination (ICI) (2 -4 ). Cryopreserved semen quality, insemination timing, cycle monitoring methods, and other factors affect the outcome of AID (5 , 6 ). Other clinical variables may also affect the outcome of AID. This study intends to investigate whether a series of clinical variables can affect the pregnancy rate of AID in order to provide individualized treatment programs for different patients.

## Materials and Methods

In this retrospective study, all patients who required AID treatment between February 2006 and November 2008 at the Reproductive Center of the Women’s Hospital, Zhejiang University School of Medicine, were enrolled in this study following informed consent. A total of 501 patients who received their first ICI cycles were included in this study. Informed consent was obtained from our hospital Ethics Committee to perform this study.

We systematically review clinical histories of the female patients and selected the first cycle of AID treatment for retrospective analysis. Clinical variables analyzed included age, years of infertility, incidence of cervicitis, urinary luteinizing hormone (LH) surge result, insemination number, uterus position, endometrial thickness and morphology, maximum follicle diameter (diameter ≥14 mm) and dominant follicle number on the day of human chorionic gonadotropin (HCG) administration as assessed by transvaginal ultrasound. We recorded all pregnancies (including live births), miscarriages or ectopic pregnancies and calculated the pregnancy rate. We excluded those patients from the analysis of each variable due to data loss or incomplete medical records.

Indications for treatment included azoospermia (98.4%), male genetic disorder (1.2%), or severe oligo-, terato-, or asthenozoospermia (0.4%). All women received a primary screening baseline evaluation for fertility potential that included a medical history, physical examination, and laboratory assessments. Hysterosalpingography and/ or laparoscopy investigations were offered to each woman within the previous two years to ensure that at least one fallopian tube was patent. Ovarian stimulation therapy using clomiphene citrate (CC), human menopausal gonadotropin (HMG), or a combination of CC/HMG were administered to those who had ovulation dysfunction (n=166); the dose was adjusted according to the ovarian response.

Ultrasound scans monitored development of the dominant follicles. When the maximal follicle diameter reached 14 mm, the urinary LH surge was examined. An injection of 5000-10000 IU HCG was administered if the urinary LH surge was positive or at least one follicle reached a diameter of >18 mm. We generally recommended that patients receive two inseminations per cycle, the first 12 ± 4 hours and the second 36 ± 4 hours after HCG injection, or on the day of a positive urinary LH surge and the following day. However, due to financial considerations or personal reasons, some patients preferred to receive a single insemination 12 ± 4 hours after HCG injection or on the day of the positive urinary LH surge.

Thawed cryopreserved semen from 98 different anonymous donors were obtained from sperm banks and used for insemination following a standard ICI protocol. The concentration and motility of all semen was checked to ensure a minimum of 5×10^6^ progressively motile spermatozoa per specimen, which has been recommended for maximum pregnancy rates (7 ). We placed 1 ml of the specimen into the insemination catheter, which was presented to the medical staff who performed standard ICI, with the semen slowly deposited directly into the cervical canal. After insemination, patients remained in the supine position with their hips elevated for 30 minutes.

Pregnancy tests were performed on day 14 after the first insemination by quantification of plasma β-hCG and confirmed 20 days later by vaginal ultrasonography. Statistical analyses were performed using the t test or Chi-square test by SPSS 13.0, where appropriate. P<0.05 was considered significant.

## Results

A total of 501 patients underwent the first AID cycles over a 34 month period, which resulted in a total of 115 pregnancies. Of pregnancies, there were 94 live births that consisted of 1 premature delivery at 28 weeks, 8 deliveries at 32 to 37 weeks, and 85 term deliveries. There were 9 sets of twins and 85 single fetuses, 20 miscarriages (18 inevitable abortions, 1 fetal malformation, and 1 hydatidiform mole) and one ectopic pregnancy. The overall pregnancy rate for the first ICI cycle was 23.0%.

The women ranged in age from 21 to 43 years. There was a significant difference in the mean age of women who achieved pregnancy (29.0 ± 3.6 years) compared to those who did not become pregnant (30.1 ± 3.9; T=2.783; p=0.006), which suggested a correlation between decreased cycle fecundity and increased age. The number of years of infertility at initial presentation ranged from 0 to 20. The mean number of years of infertility year was 5.0 ± 2.9; women with an infertility period of more than five years had a lower pregnancy rate (Chi-square=7.881, p=0.005).

A total of 151 patients were diagnosed with cervicitis and 334 had a smooth cervix. There were 16 patients who did not register the results of their cervical inspection. The pregnancy rate in patients with cervicitis (22.5%, 34/151) was not significantly different compared to those without cervicitis (22.2%, 74/334; Chi-square=0.008, p=0.930). We evaluated urinary LH surge in order to predict ovulation, however in 36 patients, the data was unavailable. Patients (n=226) with positive urinary LH surge had a pregnancy rate of 22.1% (50/226) and those (n=239) who had a negative urinary LH surge had a pregnancy rate of 25.1% (60/239), which was not significant (Chi-square=0.571, p=0.450).

Improvements in pregnancy rate have been reported by increasing the number of inseminations (8 ). Thus we compared the pregnancy rate in women who underwent one insemination (23.5%, 20/85) to those who received two consecutive inseminations (22.9%, 95/415). In this comparison there was no statistically significant difference (Chi-square=0.016, p=0.889). One patient’s record was misplaced and therefore not analyzed.

We also evaluated different uterine positions and pregnancy rate. In total, 324 patients had an anteverted uterine position, 144 had a retroverted position, and 33 did not have a record of their uterine position. Pregnancy rates were 25.3% (82/324) for anteverted and 16.7% (24/144) for retroverted, which was significant (Chi-square=4.250, p=0.039). Those with anteverted uteri had a much higher pregnancy rate.

Endometrial receptivity is a crucial factor during human reproduction. Several parameters determined by transvaginal ultrasonography, such as endometrial thickness and morphology have been proposed for the assessment of endometrial receptivity (9 -11 ). In this study, we sought to determine if endometrial thickness and morphology on the day of HCG administration could affect the pregnancy rate. Women were assigned to three groups according to endometrial thickness (<7 mm, 7-14 mm, or >14 mm). The pregnancy rates of the three groups are presented in table 1.

The 7-14 mm group had a higher pregnancy rate compared to the other two groups (Chisquare= 10.359, p=0.006). Gonen and Casper proposed that endometrial morphology under sonogram can be classified according to three types (11 ). We have renamed these as: i. type A, a multilayered ‘triple-line’ endometrium consisting of a prominent outer and central hyperechogenic line and inner hypoechogenic region; ii. type B, an intermediate isoechogenic pattern with the same reflectivity as the surrounding myometrium and a poorly defined central echogenic line; and iii. type C, an entirely homogeneous, hyperechogenic pattern that lacks a central echogenic line. The pregnancy rates of each group are presented in table 2. Those with type A endometrial morphology seemed to have a much higher pregnancy rate than the other two groups; however, there was no significant difference among the groups.

**Table 1:** Comparison of pregnancy rates of patients with different endometrial thickness


Endometrial thickness (mm)	Pregnancy n (%)	No pregnancy (n)	Chi-square	P value

**<7**	2 (7.1)	26	10.359	0.006
**7-14**	107 (25.8)	307		
**>14**	6 (10.9)	49		
**Total**	115 (23.1)	382		


Four patients had no records of their endometrial thickness.

**Table 2:** Comparison of pregnancy rates in patients with different endometrial morphology


Endometrial morphology	Pregnancy n (%)	No pregnancy (n)	Chi-square	P value

**Type A**	93 (25.1)	277	3.211	0.201
**Type B**	17 (20.7)	65		
**Type C**	5 (11.1)	40		
**Total**	115 (23.1)	382		


Four patients had no records of their endometrial thickness.

**Table 3:** Comparison of pregnancy rates in patients with different maximal follicle diameters in the natural cycle group


Maximal follicle diameter (mm)	Pregnancy n (%)	No pregnancy (n)	Chi-square	P value

**14 ~**	0 (0)	7	4.372	0.112
**16 ~**	29 (21.6)	105		
**≥18**	37 (25.7)	107		
**Total**	66 (23.2)	219		


**Table 4:** Comparison of pregnancy rates of patients with different number of follicles (diameter ≥ 14 mm) in stimulated cycle group


Follicles ≥14 mm (n)	Pregnancy n (%)	No pregnancy (n)	Chi-square	P value

**1-3**	29 (27.9)	75	3.377	0.337
**4-5**	3 (15.0)	17		
**6-9**	3 (11.5)	23		
**>9**	2 (12.5)	14		
**Total**	37 (22.3)	129		


Finally, we analyzed whether maximal follicle diameter (diameter ≥14 mm) and dominant follicle number prior to ovulation could influence pregnancy rate. For this analysis we chose the 285 women who had normal ovulation and received natural cycle inseminations to analyze the effect of maximal follicle diameter on pregnancy rate. Since these women only had one dominant follicle, the diameter was easy to measure. These women were divided into three groups according to maximum follicle diameter on the day of HCG administration (~14 mm, ~16 mm, and ≥18 mm).

The pregnancy rate of each group is shown in table 3; however we have observed no significant difference among these groups. Ovarian stimulation therapy was administered to 166 women with ovulation dysfunction. These women presented with multiple dominant follicles on the day of HCG administration. We chose these women to determine whether or not a dominant follicle number would affect pregnancy rate. The women were allocated to different groups according to the total follicle number on the day of HCG administration (1-3, 4-5, 6-9, or >9). The pregnancy rate of these groups is presented in table 4. No significant differences were observed among these groups; however, there was a trend for women with no more than three follicles to have a higher pregnancy rate. We did not record relevant data about natural cycles, the stimulating cycle of 50 patients, and the records of their follicle conditions.

## Discussion

According to traditional ethical values in China, the fundamental purpose of marriage is procreation. Couples with male infertility will endeavor to seek any method to obtain genetically linked offspring; however, AID is widely used when all other treatment options for male infertility have been unsuccessful. As a further option, *in vitro* fertilization with donor spermatozoa (IVFD) is offered to patients in whom six cycles of AID treatment have failed.

Although AID is relatively simple, less invasive, and cheaper than IVFD, the cycle success rate is slightly lower. The current cost of AID treatment in China is about 800 USD per cycle, compared to IVF which costs approximately 4000 USD per oocyte retrieval. Therefore, it is important to determine if clinical variables can affect the success rate of AID in order to enable individual therapy and improve cost-effectiveness.

The overall pregnancy rate during the first ICI cycle for all women reviewed in our study was 23.0%, which was comparable to the outcomes of other reports (20.1%) (7 ). Our results indicated that fecundity declined with increasing age, as seen in other studies (7 , 12 ). We have also observed that women with a long infertility period (more than 5 years) had a poor pregnancy rate. However, a long infertility period is often related to increased age, deteriorating ovarian function with increasing age, and mental stress in women with many years of infertility also contribute to the poor outcome (13 ).

Initially we hypothesized that inflammatory factors linked to cervicitis may alter the characteristics of cervical mucus and affect the penetration of spermatozoa; however, we observed no difference in pregnancy rates between women with and without cervicitis.

It is known that the accurate timing of impending ovulation is one of the most important factors affecting the pregnancy rate in insemination programs (6 ). In this study, we have analyzed the pregnancy rate of women with positive and negative urinary LH surges, which are commonly used to predict ovulation. No difference in pregnancy rates were observed, possibly due to the intramuscular injection of HCG that was administered to all women in this study in order to ensure ovulation at the correct time.

Although previous studies have reported significantly higher pregnancy rates in women who receive multiple ICI (8 , 14 ), we found that a single insemination was as effective as two inseminations. This result was similar to a study conducted by Lincoln et al. (15 ), which suggested that a single ICI per cycle before ovulation was sufficient in conjunction with an injection of HCG to ensure ovulation at the correct time.

In this study, we have found that women who had an anteverted uterine position had a higher pregnancy rate than those with a retroverted position. This might be due to the fact that the cervix is always positioned downwards with an anteverted uterus, ensuring that the semen and cervical mucus are in close contact during ICI treatment. Based on the results of this study, we propose that IUI would be a more suitable method of insemination in women with a retroverted uterus position.

Several studies have looked at whether Doppler sonography parameters, such as endometrial vascularity, affect the pregnancy rate or not in assisted reproductive techniques. The results of these studies are controversial (16 , 17 ). We have evaluated several transvaginal ultrasonography parameters on the day of HCG administration. According to our results, endometrial thicknesses of 7-14 mm resulted in higher pregnancy rates, but no statistical difference was found among different endometrial morphology groups. It might be attributed to the small sample size of the type C group, because many of these patients abandon the current AID cycle. The type A group seemed to have a much higher pregnancy rate than types B and C. Thus in women with an endometrial thickness of <7 mm or >14 mm, or with type C endometrial morphology, it may be necessary to weigh the advantages and disadvantages of treatment and make a reasonable decision to forsake the current AID cycle or continue with treatment. According to Hsieh et al. (18 , 19 ), the administration of low-dose aspirin to patients with a thin endometrium may improve endometrial morphology and result in a higher pregnancy rate. Additionally, an endometrial biopsy should be suggested to patients who present with poor endometrial conditions in several consecutive cycles (20 ).

No significant differences in pregnancy rate were observed between women with different maximum follicle diameters; however, the data suggested that a maximal follicle diameter less than 16 mm seemed to result in a poorer pregnancy rate.

Combined AID with ovarian stimulation has been reported to improve the pregnancy success rate (21 ), but also increase the risk of multiple pregnancies and severe complications due to ovarian hyperstimulation syndrome (OHSS). Our analysis revealed that the administration of a subtle ovarian stimulation program, which produced no more than three follicles (diameter ≥14 mm), tended to increase the pregnancy rate; however, this effect was not statistically significant. A subtle ovarian stimulation program can lower the risk in patients suffering from OHSS.

## Conclusion

An individualized treatment schedule according to the unique characteristics of each couple should be formulated for patients requiring AID therapy. Although IUI has been reported to be more effective than ICI in several studies, IUI carries a risk of endometritis, cramping, bleeding, and anaphylaxis (rarely). For young women with less than five years of infertility, ICI with or without ovarian stimulation can be recommended. In older women with more than five years of infertility (particularly cases with a retroverted uterine position), IUI with ovarian stimulation or IVFD might be adopted much earlier in order to save time and money by directly shifting to techniques which are likely to result in a higher success rate.
